# Romo1 is involved in the immune response of glioblastoma by regulating the function of macrophages

**DOI:** 10.18632/aging.102648

**Published:** 2020-01-16

**Authors:** Guan Sun, Ying Cao, Chunfa Qian, Zhengqiang Wan, Jian Zhu, Jun Guo, Lei Shi

**Affiliations:** 1 Department of Neurosurgery, Yancheng City No.1 People’s Hospital, The Fourth Affiliated Hospital of Nantong University, Yancheng, PR China; 2 Department of Ear-Nose-Throat, The Second People's Hospital of Huai’an, Huai’an Affiliated Hospital of Xuzhou Medical University, Huai’an, PR China; 3 Department of Neurosurgery, The Affiliated Brain Hospital of Nanjing Medical University, Nanjing, PR China; 4 Department of Neurosurgery, The First People's Hospital of Kunshan Affiliated with Jiangsu University, Suzhou, Jiangsu, PR China

**Keywords:** glioblastoma, immunotherapy, Romo1, macrophages, immune response

## Abstract

Reactive oxygen species (ROS) modulator 1 (Romo1) is a mitochondrial membrane protein that is essential for the regulation of mitochondrial ROS production and redox sensing. Although the physiological functions of Romo1 have been studied for the past few years, the role of Romo1 in cancer remained unclear. In this study, we found that the high expression of Romo1 is associated with the poor prognosis of glioblastoma patients. Further study revealed that Romo1 is highly expressed in macrophages, indicating that the overexpression of Romo1 may participate in the function of macrophages and contribute to the progression of glioblastoma. Through the glioblastoma mouse model, we found that the overexpression of Romo1 in bone marrow cells significantly inhibited the immune response within tumor microenvironment, and that the overexpression of Romo1 resulted in the M2 polarization of bone marrow derived macrophages (BMDMs) through mTORC1 signaling pathway. In addition, the inhibition of Romo1 combining with anti-PD-1 immunotherapy significantly improved the survival outcome of glioblastoma in mouse model. Collectively, our study highlights the important role of Romo1 in immune response especially the function of macrophages, and implicates it as a potential target of immunotherapy for glioblastoma.

## INTRODUCTION

Glioma is the most common brain neoplasms among adult worldwide, and about 50% of patients present with the most aggressive form of the disease, glioblastoma [1]. Typical therapies for glioblastoma include surgery, radiotherapy, and concomitant temozolomide-based chemotherapy, but improvements remain limited in the survival outcomes of patients [1]. Over the past decade, immunotherapy represented by PD-1/L1 immune checkpoint blockade has achieved remarkable success in several tumor types like advanced melanoma [2–5] and non-small-cell lung cancer (NSCLC) [6, 7], and has attracted considerable interest from the glioblastoma community. However, a recent clinical trial of PD-1 inhibitors in recurrent glioblastoma showed that only a small part of patients demonstrated long-term responses [8], probably due to the alterations of molecular signatures that regulate the immune tolerance within tumor microenvironment [9].

Reactive oxygen species (ROS) modulator 1 (Romo1) is a mitochondrial membrane protein that is thought to be involved in mitochondrial ROS production redox sensing in mitochondrial dynamics [10, 11]. Romo1 was considered to participate in tumor growth and invasiveness [12–14]. A recent clinical study reported that the overexpression of Romo1 predicted unfavorable prognosis and lymphatic metastasis in NLCLC [15], but the role of Romo1 in tumorigenesis still remain unclear. In our preliminary study, we found that the high expression of Romo1 is associated with the poor prognosis of glioblastoma patients and that Romo1 is highly expressed in macrophages. Through the orthotopic glioblastoma mouse model, we also found that the overexpression of Romo1 in bone marrow cells inhibited the immune response within tumor microenvironment, implicating that Romo1 may also participate in the immune tolerance of tumor. These results motivated us to characterize the role of Romo1 in macrophages and the progression of glioblastoma.

## RESULTS

### The high expression of Romo1 is associated with the poor prognosis of glioblastoma patients

**Figure 1:**
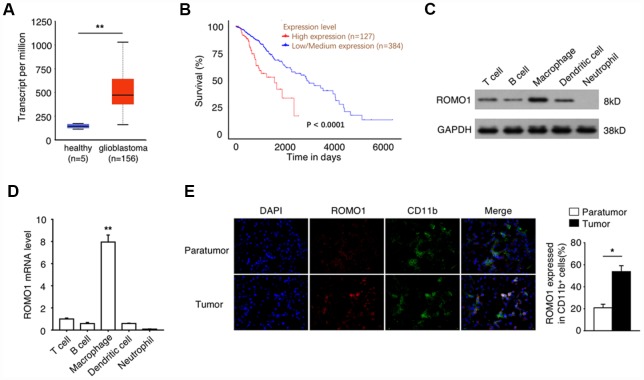
**The high expression of Romo1 is associated with the poor prognosis of glioblastoma patients.** (**A**) The mRNA levels of Romo1 in the tumor tissues of glioblastoma patients (n=156) and the healthy brain tissues (n=5) were compared. The mRNA levels were compared between the samples with RNA-Seq data published in TCGA database. (**B**) The survival curves of the glioblastoma patients with high expression (n=127) and low expression (n=384) of Romo1. The mRNA levels were compared between the samples with mRNA microarray data published in TCGA database. (**C**) The protein levels of Romo1 in different types of patient-derived immune cells were detected by western blotting. (**D**) The mRNA levels of Romo1 in different types of patient-derived immune cells were analyzed by RT-qPCR. (**E**) The tissue sections of the paratumors and tumors from glioblastoma patients were analyzed by immunofluorescence with DAPI, Romo1 and CD11b antibodies. The CD11b positive cells and the Romo1 positive cells were quantified and statistically analyzed from three independent experiments. *, P < 0.05; **, P < 0.01.

To reveal the relationship between Romo1 expression and the progression of glioblastoma, we extracted the mRNA levels of Romo1 in the tumor tissues of glioblastoma patients with RNA-Seq data (n=156) from the Glioblastoma Multiforme dataset of TCGA database. We found that the mRNA levels in glioblastoma samples were significantly higher than the mRNA levels of Romo1 in the normal brain samples (n=5, p<0.01, Figure 1A). Further we asked whether the expression levels influence the prognosis of the glioblastoma patients. We analyzed the survival information of the patients with the mRNA microarray data (n=511) from the Glioblastoma Multiforme dataset of TCGA database, and found that the overall survival of the patients with high expression of Romo1 (n=127, top 25% in rank) was significantly lower than the patients with low expression of Romo1 (n=384, bottom 75% in rank, p<0.0001, Figure 1B), indicating that the high expression of Romo1 may participate in the progression of glioblastoma.

### Romo1 is expressed with a relatively high level in tumor-associated macrophages

Then we asked whether the high expression of Romo1 occur in the immune cells within tumor microenvironment. Firstly we compared the protein and the mRNA levels of Romo1 in the patient-derived T cells, B cells, neutrophils, macrophages and dendritic cells, and found that Romo1 is expressed in T cells, B cell, macrophages and dendritic cells, but not neutrophils (Figure 1C and 1D). Interestingly, we also found that the expression level of Romo1 is relatively higher in macrophages than other expressed cell types (Figure 1C and 1D). To further confirm the expression of Romo1 in tumor-associated immune cells, we performed the immunofluorescence analysis and found that the expression of Romo1 in CD11b+ cells within tumor tissues was significantly higher than paratumor tissues (Figure 1E). Considering that CD11b mainly express in monocytes/macrophages and neutrophils and that the expression of Romo1 in neutrophils was close to negative, this result suggested that Romo1 expressed in a relatively high level in tumor-associated macrophages.

### The overexpression of Romo1 in bone marrow cells promoted the progression of glioblastoma and suppressed the T cell response in mouse model

**Figure 2:**
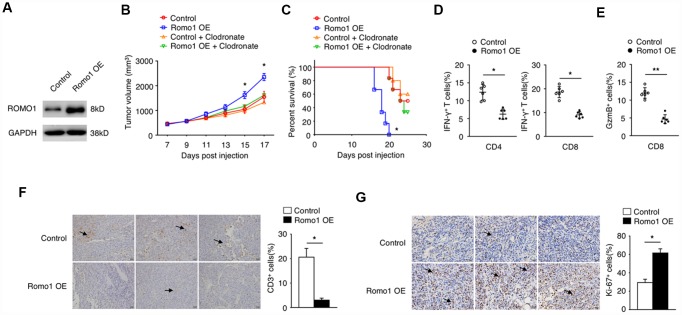
**The overexpression of Romo1 in bone marrow cells dampened the immune response in glioblastoma.** (**A**) The overexpression of Romo1 in the bone marrow cells transfected with Romo1-lentivirus (the right lane) was validated by western blotting. The protein sample of the bone marrow cells transfected with control lentivirus (the left lane) was used as control. (**B**, **C**) The tumor growth curve (**B**) and the survival curve (**C**) of the mice receiving the control bone marrow allografts alone (n=6) or in combination of clodronate liposomes (200ul of each mouse every 3 days, n=6),, and the mice receiving the Romo1-overexpressed bone marrow allografts alone (n=6) in combination of in combination of clodronate liposomes (200ul of each mouse every 3 days, n=6) after orthotopic injection of GL261 cells. (**D**) The tumor sections were analyzed by immunohistochemistry with CD3 antibody, and the infiltrating CD3+ T cells around tumors were quantified and statistically analyzed. Arrows indicate CD3+ T cells. (**E**) The frequencies of IFN-γ-producing CD4+ or CD8+ T cells in tumors of both groups were determined by flow cytometry and statistically analyzed. (**F**) The frequencies of GzmB-producing CD4+ or CD8+ T cells in tumors of both groups were determined by flow cytometry and statistically analyzed. (**G**) The tumor sections from both groups were analyzed by immunohistochemistry with Ki-67 antibody and statistically analyzed from three independent experiments. Arrows indicate the Ki-67+ cells. *, P < 0.05; **, P < 0.01.

To further study the role of immune cell expressed Romo1 in the tumorgenesis of glioblastoma, we transplanted the Romo1-lentivirus transduced or the control bone marrow cells into the recipient mice (Figure 2A–2C). Firstly, we validated the overexpression of Romo1 in the Romo1-lentivirus transduced bone marrow cells (Figure 2A). On this basis, we orthotopically injected the Romo1-overexpressed and the control mice with GL261 cells. The follow-up observation showed that the growth of the tumors in mice bearing the Romo1-overexpressed bone marrow cells was significantly faster than the control mice (p<0.05 at Day 15 or 17 after injection, Figure 2B). Although both groups of mice died in succession after GL261 injection, the mice bearing the Romo1-overexpressed bone marrow cells had a significantly shorter disease latency compared with the control mice (p<0.05, Figure 2C) To further examine whether the difference between the control and the Romo1-overexpressed group of mice was macrophage-dependent, we injected both group of mice with the clodronate liposomes, one kind of macrophage-depleting agents, through the tail vein every 3 days (Figure 2B and 2C). We found that the clodronate liposomes significantly inhibited the in-vivo growth of GL261 cells (p<0.05 at Day 15 or 17 after injection, Figure 2B) and prolonged the survival curve of the mice transplanted with Romo1-overexpressed bone marrow cells (p<0.05, Figure 2C), indicating that the overexpression of Romo1 in bone marrow cells, mainly in the macrophages, promoted the progression of glioblastoma.

We sacrificed the moribund glioblastoma mice to examine the phenotype within tumor microenvironment. The results showed that the numbers of CD3+ cells in the tumors of the mice bearing the Romo1-overexpressed bone marrow cells were significantly less than the control mice (p<0.05, Figure 2D). Flow cytometry analysis showed that the ratio of IFN-γ-producing CD4+ or CD8+ cells or GzmB+/CD8+ cells in the tumors of the mice bearing the Romo1-overexpressed bone marrow cells were significantly lower than the control mice (p<0.05 and p<0.01 respectively, Figure 2E and 2F. The immunochemistry analysis showed that the expression of Ki-67 in the tumors of the mice bearing the Romo1-overexpressed bone marrow cells were significantly stronger than the control mice (Figure 2G). These indicated that the overexpression of Romo1 in bone marrow cells could suppress the T cell response in glioblastoma.

### The overexpression of Romo1 promoted the accumulation of cellular ROS production in macrophages

**Figure 3:**
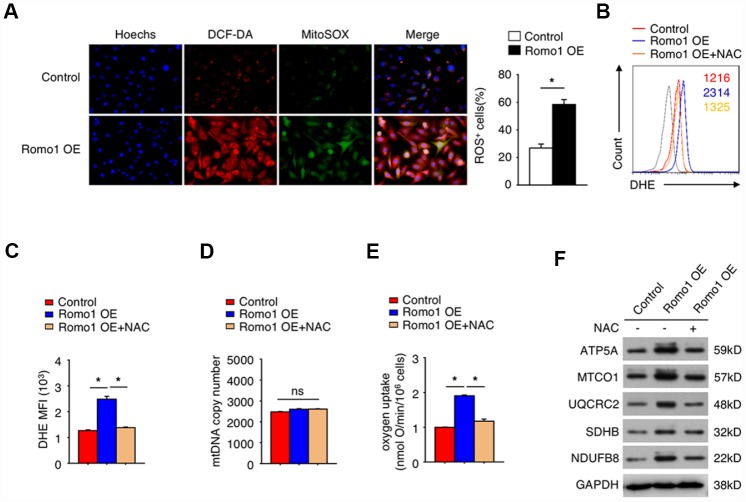
**The overexpression of Romo1 promoted the accumulation of ROS and results in mitochondrial dysfunction in macrophages.** (**A**) The control and the Romo1-overexpressed macrophages were analyzed by immunofluorescence with DCF-DA and MitoSOX staining to detect the levels of cytoplasmic and mitochondrial ROS. The ROS+ cells were counted under microscopy and statistically analyzed. (**B**) The levels of DHE in control and Romo1-overexpressed macrophages (with or without treatment of 5mM NAC) were analyzed by flow cytometry. (**C**) The mean fluorescence intensity (MFI) of DHE in flow cytometry was respectively quantified and statistically analyzed. (**D**) The statistical analysis of the mtDNA copy number in control and Romo1-overexpressed (with or without treatment of 5mM NAC) macrophages. (**E**) The statistical analysis of the oxygen uptake rate in control and Romo1-overexpressed (with or without treatment of 5mM NAC) macrophages. (**F**) The expression of the indicated proteins was examined by western blotting in control and Romo1-overexpressed (with or without treatment of 5mM NAC) macrophages. Data are representative of at least three independent experiments and are presented as mean ± SD. ns, not significant; *, P < 0.05; **, P < 0.01.

It is well known that Romo1 participates in mitochondrial ROS production. To confirm the role of Romo1 in the ROS production in macrophages, we constructed the Romo1-overexpressed BMDMs and examined the ROS levels with DCF-DA and mitoSOX probes. In compared with the control BMDMs, the Romo1-overexpressed BMDMs presented significantly higher ability of probe combination (Figure 3A).

Moreover, we used the dihydroethidium (DHE) to detect the cellular ROS levels. As predicted, the DHE staining intensity of Romo1-overexpressed BMDMs was significantly stronger than the control BMDMs (Figure 3B and 3C). Although the mitochondrial DNA copy number was not influenced when overexpression of Romo1 (Figure 3D), the oxygen uptake was significantly enhanced (Figure 3E). When N-acetyl-cysteine (NAC, a ROS scavenger) was added into Romo1-overexpressed BMDMs, the DHE staining intensity or the oxygen uptake was rescued (Figure 3B, 3C and 3E).

We also examined the expression of the proteins associated with mitochondrial ROS production, including ATP5A, MTCO1, UQCRC2, SHDB and NDUFB8, and found that the levels of these proteins was significantly upregulated when overexpression of Romo1 in BMDMs, but their expression levels were rescued after addition of NAC into the Romo1-overexpressed BMDMs (Figure 3F). These results suggest that the overexpression of Romo1 promoted the accumulation of ROS production and may result in the mitochondrial dysfunction in macrophages.

### The overexpression of Romo1 increased the anti-inflammatory function and promoted the cellular metabolism reprogramming of macrophages

**Figure 4:**
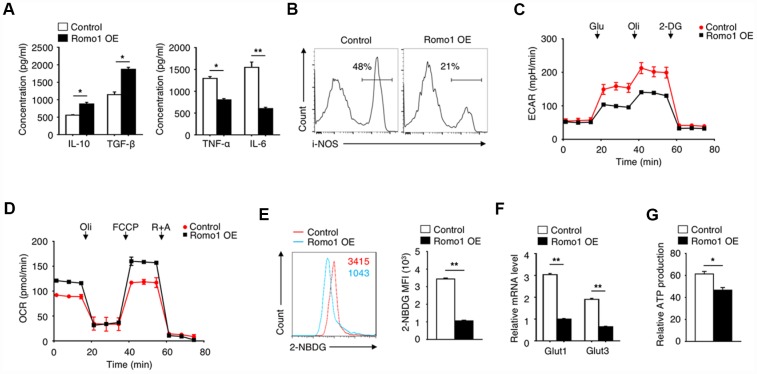
**The overexpression of Romo1 triggered the metabolic reprogramming in macrophages.** (**A**) The levels of IL-10, TGF-β, TNF-α and IL-6 were analyzed by ELISA in control and Romo1-expressed macrophages. (**B**) The levels of iNOS were analyzed by flow cytometry in control and Romo1-overexpressed macrophages. (**C**) The extracellular acidification rates (ECAR) of control and Romo1-overexpressed macrophages were measured under basal conditions followed by the sequential addition of 10nM glucose (Glu), 0.5μM oligomycin (Oli) and 100mM 2-deoxyglucose (2-DG). (**D**) The oxygen consumption rates (OCR) of control and Romo1-overexpressed macrophages were measured under basal conditions followed by the sequential addition of 0.5μM oligomycin (Oli), 1μM carbonyl cyanide p-trifluoromethoxy-phenylhydrazone (FCCP) and 1μM rotenone and antimycin A (R+A). (**E**) The levels of 2-NBDG were analyzed by flow cytometry in control and Romo1-overexpressed macrophages. The MFIs of 2-NBDG were quantified and statistically analyzed. (**F**) The mRNA levels of Glut1 and Glut3 were determined by qRT-PCR in control and Romo1-overexpressed macrophages. (**G**) The relative ATP levels of the control and Romo1-overexpressed macrophages. Data are representative of at least three independent experiments and are presented as mean ± SD. ns, not significant; *, P < 0.05; **, P < 0.01.

When investigating the role of Romo1 in macrophage-mediated inflammation, we found that the overexpression of Romo1 increased the production of anti-inflammatory cytokines (IL-10 and TGF-β) and decreased the level of pro-inflammatory cytokines (TNF-α and IL-6) in macrophages, which indicated the anti-inflammatory phenotype of macrophages (Figure 4A). We also examined the expression of iNOS, which can be induced in response to cytokines, in the Romo1-overexpressed and the control macrophages by flow cytometry, and found that the expression levels of Romo1 in Romo1-overexpressed macrophages is lower than the control cells (Figure 4B), indicating that the overexpression of Romo1 increased the anti-inflammation function of macrophages.

To further examine the effect of Romo1 overexpression on the cellular metabolism status, we compared the extracellular acidification rate (ECAR) or the oxygen consumption rate (OCR) between the Romo1-overexpressed BMDMs and the control BMDMs under different mitochondrial stress. Compared with the control BMDMs, the ECAR was promoted while the OCR was inhibited in the Romo1-overexpressed BMDMs (Figure 4C and 4D), suggesting that the overexpression of Romo1 promoted the glycolysis but inhibited the oxidative phosphorylation in BMDMs.

In addition, we used 2-NBDG to measure the ability of cellular glucose uptake and found that the incorporation of 2-NBDG in Romo1-overexpressed BMDMs was significantly less than the control cells (Figure 4E). The mRNA levels of Glut1 and Glut3 in Romo1-overexpressed BMDMs were significantly downregulated compared with the control cells (Figure 4F). Besides, the ATP production in Romo1-overexpressed BMDMs was significantly lower than the control cells (Figure 4G). These results suggest that the overexpression of Romo1 promoted the cellular metabolism reprogramming in macrophages.

### The overexpression of Romo1 resulted in M2 polarization of macrophages through mTORC1 signaling pathway

**Figure 5:**
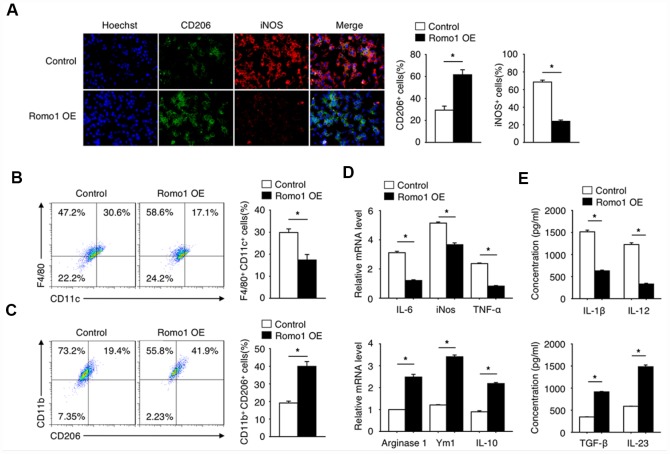
**The overexpression of Romo1 promoted macrophage polarization toward M2 phenotype.** (**A**) The control and Romo1-overexpressed macrophages were analyzed by immunofluorescence with CD206 and iNOS antibodies. The CD206+ and iNOS+ cells were quantified and statistically analyzed. (**B**, **C**) The control and Romo1-overexpressed macrophages were analyzed by flow cytometry with M1 markers (CD11c and F4/80) and M2 markers (CD206 and CD11b). The double positive cells were respectively quantified and statistically analyzed. (**D**) The mRNA levels of M1-related genes (IL-6, iNOS, TNF-α) and M2-related genes (Arginase 1, Ym1, IL-10) were determined by RT-qPCR in control and Romo1-overexpressed macrophages. (**E**) The production of IL-1β, IL-12, TGF-β or IL-23 was respectively analyzed by ELISA in control and Romo1-overexpressed macrophages. Data are representative of at least three independent experiments and are presented as mean ± SD. ns, not significant; *, P < 0.05; **, P < 0.01.

Since the polarization of macrophages plays an important role in tumorigenesis, we then asked whether the upregulation of Romo1 influences the status of macrophage polarization. Through the immunofluorescence experiment, we found that the expression of CD206 (M2 marker) in Romo1-overexpressed BMDMs was significantly upregulated, while the expression of iNOS (M1 maker) was significantly downregulated (Figure 5A), when compared with the control cells. The flow cytometry analysis also presented that the ratio of CD11b+/CD206+ cells in the Romo1-overexpressed BMDMs was significantly higher than the control cells, while the ratio of F4/80+/CD11c cells was significantly lower (Figure 5B and 5C). The examination of mRNA and protein levels of M1 markers (including IL-6, Nos2, TNF-α or IL-23) or M2 makers (Arginase1, Ym1, IL-10 or TGF-β, IL-23) all indicated that the overexpression of Romo1 promoted M2 polarization of macrophages (Figure 5D and 5E).

**Figure 6:**
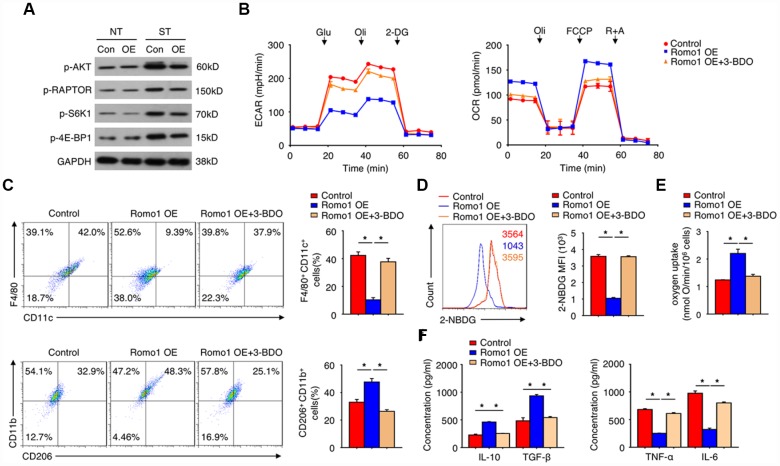
**The overexpression of Romo1 promoted M2 polarization through mTORC1 signaling pathway in macrophages.** (**A**) The expression of the indicated proteins was examined by western blotting in the control and Romo1-overexpressed macrophages with or without treatment of LPS (NT: without treatment; ST: LPS stimulation). (**B**) The ECAR and OCR of control and Romo1-overexpressed (with or without treatment of 60μM 3-BDO) macrophages were measured under basal conditions followed by the indicated treatment. (**C**) The control and Romo1-overexpressed (with or without treatment of 60μM 3-BDO) macrophages were respectively analyzed by flow cytometry with M1 or M2 markers. (**D**) The levels of 2-NBDG were analyzed by flow cytometry in control and Romo1-overexpressed (with or without treatment of 60μM 3-BDO) macrophages. (**E**) The oxygen uptake rates were measured and quantified in the control and Romo1-overexpressed (with or without treatment of 60μM 3-BDO) macrophages. (**F**) The production of IL-10, TGF-β, TNF-α or IL-6 was respectively analyzed by ELISA in control and Romo1-overexpressed (with or without treatment of 60μM 3-BDO) macrophages. Data are representative of at least three independent experiments and are presented as mean ± SD. ns, not significant; *, P < 0.05; **, P < 0.01.

To reveal the underlying molecular mechanisms, we performed a series of signaling screening (data not shown), and found that in response to LPS stimulation, the overexpression of Romo1 inhibited the activation of mTORC1 pathway including the phosphorylation of AKT, RAPTOR, S6K1 and 4E-BP1 (Figure 6A). When 3-BDO was added to rescue the activation of mTORC1 pathway, the promotion effects of glycolysis, M2 polarization and oxygen uptake or the inhibition effect of glucose uptake induced by Romo1 overexpression were significantly compromised (Figure 6B–6F). These results indicated the mTORC1 signaling pathway contributes, in a large extent, to the effect of Romo1 upregulation in macrophages.

### The combination of Romo1 inhibition and PD-1 blockade significantly improved the survival outcome of glioblastoma in mouse model

**Figure 7:**
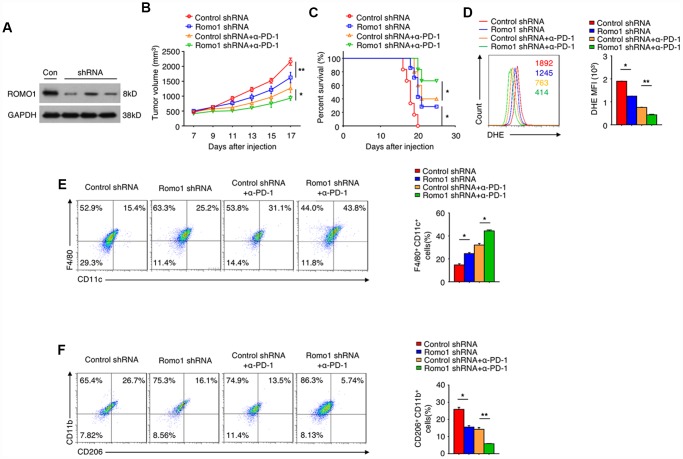
**The inhibition of Romo1 enhanced the efficacy of anti-PD-1 immunotherapy in glioblastoma.** (**A**) The Romo1 shRNA efficiency in bone marrow cells was determined by western blotting. (**B**, **C**) The tumor growth curve (**B**) and the survival curve (**C**) of the mice receiving control or Romo1 knockdown bone marrow allografts (with or without treatment of PD-1 antibody on Day 7, 10 and 13) and orthotopic injection of GL261 cells. (**D**) The DHE levels were analyzed by flow cytometry in control and Romo1-overexpressed (with or without treatment of PD-1 antibody) macrophages. (**E**, **F**) The control and Romo1-overexpressed (with or without treatment of PD-1 antibody on Day 7, 10 and 13) macrophages were respectively analyzed by flow cytometry with M1 (**E**) or M2 (**F**) markers. Data are representative of at least three independent experiments and are presented as mean ± SD. ns, not significant; *, P < 0.05; **, P < 0.01.

We further investigated the potential of Romo1 as the target of glioblastoma immunotherapy. Firstly we transplanted the recipient mice with the Romo1-shRNA transduced bone marrow cells (Figure 7A), and then orthotopically injected with GL261 cells. We found that when the expression of Romo1 in bone marrow cells was knocked down, the tumor growth of glioblastoma was significantly inhibited (Control shRNA vs. Romo1 shRNA, p<0.01, Figure 7B), while the disease latency was significantly prolonged (Control shRNA vs. Romo1 shRNA, p<0.05, Figure 7C). Besides, when the expression of Romo1 in BMDMs (originated from the glioblastoma-bearing mice) was knocked down, the ROS level was significantly inhibited (Control shRNA vs. Romo1 shRNA, p<0.05, Figure 7D), and the M1 polarization was significantly promoted while the M2 polarization was inhibited (Control shRNA vs. Romo1 shRNA, p<0.05, Figure 7E).

In addition, we treated the groups of control-shRNA and Romo1-shRNA glioblastoma mice with PD-1 antibody, and found that the combination of PD-1 blockade and Romo1 inhibition significantly enhanced the inhibitory effect on the progression of glioblastoma (Control-shRNA+anti-PD-1 vs. Romo1-shRNA+anti-PD-1, p<0.05, Figure 7B and 7C). Moreover, the tendency of ROS inhibition, M1 promotion and M2 inhibition on BMDMs was further enhanced with the combination of PD-1 blockade and Romo1 inhibition (Control-shRNA+anti-PD-1 vs. Romo1-shRNA+anti-PD-1, p<0.01, Figure 7D; Control-shRNA+anti-PD-1 vs. Romo1-shRNA+anti-PD-1, p<0.05, Figure 7E; Control-shRNA+anti-PD-1 vs. Romo1-shRNA+anti-PD-1, p<0.01, Figure 7F). The synergistic effect also suggested that the inhibition of Romo1 in bone marrow cells could enhance the efficacy of anti-PD-1 immunotherapy for glioblastoma.

## DISCUSSION

In our preliminary study, we found that the overexpression of Romo1 is associated with the poor prognosis of glioblastoma patients. Although several studies indicated that Romo1 may directly promote the tumor growth or metastasis [12–14], our study showed that the overexpression of Romo1 seemed to specifically occurred in monocytes/macrophages within tumor microenvironment of glioblastoma. To confirm the relationship between the Romo1 overexpression of macrophages and the progression of glioblastoma, we constructed the mouse model with Romo1 overexpression in bone marrow cells and on this basis established the glioblastoma mouse model. We found that the overexpression of Romo1 in bone marrow cells significantly inhibited the immune response within tumor microenvironment and promoted the progression of glioblastoma, suggesting that the overexpression of Romo1 in macrophages may be an important mechanism of immune tolerance for glioblastoma.

As a mitochondrial membrane protein, Romo1 was identified to play an important role in the regulation of mitochondrial ROS production and redox sensing [11]. Indeed in our study, we found that the overexpression of Romo1 could promote the accumulation of ROS and result in mitochondrial dysfunction in BMDMs. The mechanisms of Romo1 in mitochondrial function remained elusive for a long time. Two recent studies pointed out that Romo1 may function as a mitochondrial nonselective cation channel [16] or a constituent of the human presequence translocase [17]. Whether the ROS accumulation induced by Romo1 overexpression in macrophages depends on its functions of cation channel or translocase requires further study in the future.

Macrophages can be categorized into M1 and M2 macrophages based on their distinct functional abilities in response to microenvironmental stimuli [18, 19]. The macrophages with M2 phenotype are also considered as one type of myeloid-derived suppressor cells (MDSCs) within tumor microenvironment. This subset of MDSCs could inhibit the T cell function through production of arginase (ARG), inducible nitric oxide synthase (iNOS) and immunosuppressive cytokines such as TGF-β and IL-10 [20, 21]. In our study, we found that the overexpression of Romo1 promoted macrophage polarization toward M2 phenotype, which may be an important mechanism leading to the suppressed T cell response within tumor microenvironment in those mice transduced with Romo1-overexpressed bone marrow cells. Furthermore, our study showed that the combination of Romo1 inhibition and PD-1 blockade significantly improved the survival outcome of glioblastoma in mouse model, implicating that Romo1 may have an important role in regulating the crosstalk between tumor-associated macrophages and T cells.

In the in-vitro experiments, we also found that the effect of Romo1 overexpression on macrophage polarization is through the mTORC1 signaling pathway, and is associated with the metabolic regulation in macrophages. Although the role of mTOR signaling pathway on macrophage polarization remains controversial [22], our study suggests that the activation of mTORC1 may promote the M1 polarization of macrophages and antagonize the effect of Romo1 overexpression. It is valuable to establish a knockout system applied in monocyte/macrophage lineage in the future to elucidate the mechanisms of how Romo1 regulates the mTROC1 signaling pathway and the polarization of macrophages.

## MATERIALS AND METHODS

### Patients

Fresh glioma specimens and paratumor tissues samples were obtained from patients undergoing surgery in Yancheng City No.1 People’s Hospital between 2011-2016. The histopathologic diagnoses were performed by the pathologists according to WHO criteria. The study was approved by the Institutional Ethics Committee of Yancheng City No.1 People’s Hospital. The research was performed according to the government policies and Helsinki Declaration. All participants signed the written informed consent. Clinical and pathological characteristics were summarized in Table 1.

**Table 1:** Clinical and pathological characteristics.

**Feather**	**Patients**
All cases	27
Age, years	
<50	19
>=50	8
Gender	
Male	17
Female	10
WHO grade	
I	5
II	12
III	7
IV	3
KPS	
<90	15
>=90	12

### The isolation or culture of the patient-derived cells

Respectively, we used the Dynabeads Human CD3 Kit (Invitrogen, #11365D), Dynabeads Human B Cells Kit (Invitrogen, #11351D), Dynabeads Human CD15 Kit (Invitrogen, #11137D), Dynabeads Human DC Enrichment Kit (Invitrogen, #11308D) and Dynabeads Human Monocytes Kit (Invitrogen, #11350D) to isolate the T cells, B cells, neutrophils, dendritic cells and monocytes from the patients’ peripheral blood. All the experiments were performed according to the protocols of the manufactures. To obtain the patient-derived macrophages, we cultured the isolated monocytes in 1640 medium (GIBCO, #21875) with 10% FBS (Biological Industries, #04-001-1A) and 50ng/ml Recombinant Human GM-CSF (Peprotech, #300-25) for 5 days.

### Cell culture

GL261 glioma cell line was purchased from the American Type Culture Collection (ATCC), and were cultured in DMEM/F12 medium (GIBCO, #11320) with 10% FBS (Biological Industries, #04-001-1A). To obtain the bone marrow derived macrophages (BMDMs), fresh mouse bone marrow cells from female C57BL/6 mice were cultured in 1640 medium (GIBCO, #21875) with 10% FBS and 50ng/ml M-CSF (Peprotech, #315-02) for 5 days, and then the cell morphology was observed under microscopy.

### Plasmid construction and lentivirus packaging

The gene sequence of Romo1 was retrieved from NCBI database (Accesion number: NM_001164216.1). For Romo1 overexpression experiment, the ORF sequence of Romo1 was cloned into pLVX-ZsGreen plasmid. For Romo1-shRNA, the paired small hairpin sequence (5’-TGCAGAGTGGCGGCACGTT-3’) was cloned into pLKO.1 plasmid. The vectors were used as control. The lentivirus was packaged using the PSPAX2-PMD2G system, and the titer was determined before use.

### Mouse bone marrow transplantation

Before transplantation, bone marrow cells from the sacrificed donor mice (C57BL/6, male, 6-8 weeks) were collected and transfected with control or Romo1-overexpressed lentivirus for 48 hours. After receiving 7.5Gy of radiation, the recipient mice (C57BL/6, female, 6-8 weeks) were injected with 4×10^5^ of transfected bone marrow cells per mouse through the tail vein. The recipient mice were carefully looked after until recovery for further experiments. All the experiments were approved and supervised by the Animal Welfare and Ethics Committee of Yancheng City No.1 People’s Hospital.

### Glioblastoma mouse model

The construction of the orthotopic glioblastoma mouse model was referred to the previous study [23]. Firstly, the mice transplanted with the control or Romo1-overexpressed bone marrow were anesthetized with 0.75% pentobarbital sodium (50mg per kg body weight) and performed with craniotomy. Each mouse was carefully injected with 1×10^5^ of suspension GL261 cells through bregma with micro pump (2.5μl/min). Then, the injection hole was clogged with bone wax, and the incision was carefully stitched. Each group included at least 5 mice. The orthotopic tumor size was monitored by MRI, and the survival condition of each mouse was recorded after surgery.

For the treatment with clodronate liposomes (Liposoma BV) in the glioblastoma mouse model, 100μl of clodronate liposomes per mouse were injected through the tail vein every 3 days from Day 7 post orthotopic injection of GL261 cells. For the treatment with PD-1 antibody in the glioblastoma mouse model, the PD-1 antibody (RMP1-14, Bio X Cell) was intraperitoneally injected at 5 mg/kg every 3 days from Day 7 post orthotopic injection of GL261 cells. All the experiments were approved and supervised by the Animal Welfare and Ethics Committee of Yancheng City No.1 People’s Hospital.

### Western blotting

Cells were lysed with 1% SDS cell lysis buffer, and then were subjected to electrophoresis by SDS-PAGE for further immunoblot. Romo1 antibody was purchased from Novus (#NBP2-45607). MTCO1 and SHDB antibodies were purchased from Abcam (#ab14705 and #ab178423). p-AKT(Ser473), p-RAPTOR(Ser792), p-S6K1(Thr389) and p-4E-BP1(Ser65) antibodies were purchased from CST. Other primary antibodies that target ATP5A, UQCRC2, NDUFB8, GAPDH and β-Actin were purchased from Proteintech (Wuhan, China). The immune bonds were exposed using chemiluminescence (Cell Signaling Technology).

### ELISA

The ELISA kits for the detection of IL-10, TGF-β, TNF-α, IL-6, IL-1β, IL-12 or IL-23 levels within cells were purchased from R&D systems. The experiment was performed according to the manual protocol. All samples, standards, and controls were assayed in duplicate. The absorbance was read by Biotek ELx800 (Thermofisher, CA, USA).

### Immunofluorescence

To examine the expression of Romo1 in different types of cells within tissues, the normal brain and paratumor tissues collected from the glioblastoma mouse model were fixed in 4% paraformaldehyde and made into slices. After blocking in 3% BSA, the samples were stained with Romo1 antibody (Novus, #NBP2-45607) and CD11b antibody (CST, # 46512), followed by FITC or Alexa Fluor 700 conjugated secondary antibodies (CST).

To analyze the protein expression in BMDMs, the control or Romo1-overexpressed BMDMs of 2×10^4^ cells/cm^2^ density were seeded into the 12-well culture plates with coverslips on the bottom. After cell attachment for 24 hours, the coverslips were collected and fixed with 4% paraformaldehyde. The cells were stained with DCF-DA (Invitrogen, #D399) and MitoSOX (Invitrogen, #M36008); or with CD206 antibody (Novus, #NBP1-90020) and iNOS antibody (BD, #610431) followed by FITC or Alexa Fluor 700 conjugated secondary antibodies (CST).

### Immunochemistry

The tissue samples were collected from the mouse model and fixed in 4% paraformaldehyde overnight at room temperature. After dehydration and embedding in paraffin, the samples were sliced into 5-8 μm thickness and transferred onto glass slides. The slices were stained with CD3 antibody (CST, #99940) or Ki-67 antibody (CST, #12202) at 4°C overnight and then with biotinylated secondary antibody. After incubation with Sav-HRP conjugates, the sections were applied with DAB substrate for color development and observed under microscopy.

### Flow cytometry analysis

To examine the levels of ROS in BMDMs, 10μM dihydroethidium (DHE) was added into the culture medium for 1 hour. The cells were digested with 0.25% trypsin and suspended with pre-cold PBS for flow cytometry analysis. To detect the uptake ability of glucose, 500μM 2-NBDG was added into the culture medium for 4 hour and then digested for flow cytometry analysis. To determine the frequencies of IFN-γ+ or GzmB+ cells in CD4+ or CD8+ T cells, the suspension tumor cells were fixed, permeabilized and then stained with IFN-γ or GzmB antibody (Abcam, followed by staining with fluorophore-conjugated secondary antibodies), and CD4 or CD8 antibody (eBioscience) for flow cytometry analysis. To examine the polarization of macrophages, 3×10^5^ of BMDMs were fixed, permeabilized and then stained with iNOS, CD11c, F4/80, CD11b or CD206 antibodies (eBioscience) for flow cytometry analysis. All these data were analyzed by FlowJo software.

### RT-qPCR

The RT-PCR experiments were performed using the GoTaq qPCR System (Promega, #A6001) on the ABI 7500 Real-Time PCR System. Each sample was examined in triplicate from three independent experiments. The PCR primers for Romo1, Glut1, Glut3, IL-6, NOS2, TNF-α, Arginase1, Ym1, IL-10 and β-Actin were designed and synthesized by Sangon Biotech. Related primer sequence was provided in Table 2.

**Table 2:** Primers for real-time PCR.

**Genes**	**Forward (5′-3′)**	**Reverse (5′-3′)**
ROMO1	GAGAGACGTAGAGCTGAGCGAC	CCGGCATCTCACCTCGC
GLUT1	TACACCCCAGAACCAATGGC	CCCGTAGCTCAGATCGTCAC
GLUT3	CGGAATGCTCTTCCCCTCAG	AGTCGGCTGGTTTGTGAGAG
IL-6	GTCCTTCCTACCCCAATTTCCA	CGCACTAGGTTTGCCGAGTA
iNOS	TGCCAGGGTCACAACTTTACA	CAGCTCAGTCCCTTCACCAA
TNF-α	ATGGCCTCCCTCTCATCAGT	TTTGCTACGACGTGGGCTAC
Ym1	GGGCCCTTATTGAGAGGAGC	CCAGCTGGTACAGCAGACAA
IL-10	GGTTGCCAAGCCTTATCGGA	GGGGCATCACTTCTACCAGG
Arginase1	ACATTGGCTTGCGAGACGTA	ATCACCTTGCCAATCCCCAG

### Mitochondrial stress test assay

This assay was performed according to the previous reports [24, 25]. The BMDMs were seeded in Seahorse cell culture plates with basal medium, and then added in sequence with 10nM glucose (Glu), 0.5μM oligomycin (Oli) and 100mM 2-deoxyglucose (2-DG); or with 0.5μM oligomycin (Oli), 1μM carbonyl cyanide p-trifluoromethoxy-phenylhydrazone (FCCP) and 1μM rotenone and antimycin A (R+A). The extracellular acidification rate (ECAR) or the oxygen consumption rate (OCR) was then detected by using the Seahorse XF Extracellular Energy Analyzers.

### Measurement of ATP concentration, mtDNA copy number and oxygen uptake rates

The cellular ATP concentration was determined using the ATP detection kit (Beyotime). The detection of mtDNA copy number was performed according to the previously described [26]. As for the measurement of oxygen uptake rates, MitoXpress Intra kit (Luxcel Biosciences) was used. All the experiments were performed according to the manufacturer’s instructions.

### Statistical analysis

The information of the glioblastoma patients were extracted from the TCGA database, including mRNA levels from RNA-Seq data or microarray and the prognosis of the related patients. All the data were analyzed by Graphpad software. Student-t test was used to compare the difference between groups. The survival was analyzed by Kaplan-Meier method. The asterisks * and ** respectively represent p<0.05 and p<0.01.
